# Curcumin-photosensitized nanocapsules: biocompatibility and antimicrobial evaluation in primary tooth dentin contaminated with *Streptococcus mutans*

**DOI:** 10.3389/fcimb.2025.1614363

**Published:** 2025-09-08

**Authors:** Michelle Cristina Erckmann, Aline Almeida, Diogo Dominguini, Daniela Becker, Josiane Khun Rutz, Dachamir Hotza, Abhishek Parolia, Vanessa Valgas Dos Santos, Michael Ramos Nunes, Cleonice Gonçalves Da Rosa, Anelise Viapiana Masiero

**Affiliations:** ^1^ Multi-User Laboratory, Graduate Program in Environment and Health, Planalto Catarinense University, Lages, SC, Brazil; ^2^ Laboratory of Plasmas, Films, and Surfaces, Santa Catarina State University (UDESC), Joinville, SC, Brazil; ^3^ Laboratory of Experimental Pathophysiology, Graduate Program in Health Sciences, University of Extreme South of Santa Catarina (UNESC), Criciúma, Brazil; ^4^ Center of Biomaterials Development and Control, Faculty of Dentistry, Federal University of Pelotas, Pelotas, Rio Grande do Sul, Brazil; ^5^ Graduate Program in Chemical Engineering (PosENQ), Federal University of Santa Catarina (UFSC), Florianópolis, SC, Brazil; ^6^ Department of Chemical and Food Engineering (EQA), Federal University of Santa Catarina (UFSC), Florianópolis, SC, Brazil; ^7^ Department of Endodontics, College of Dentistry and Dental Clinics, University of Iowa, Iowa City, IA, United States; ^8^ Federal Institute of Santa Catarina, Lages, SC, Brazil

**Keywords:** dental caries, curcumin, nanotechnology, photodynamic therapy, pediatric dentistry

## Abstract

**Introduction:**

Dental caries is a multifactorial disease with high prevalence, particularly in vulnerable populations, where *Streptococcus mutans* contributes to lesion progression via acid production and biofilm formation. Minimally invasive strategies, such as photodynamic therapy (PDT) combined with advanced delivery systems, offer promising alternatives for caries management.

**Methods:**

Zein-based nanocapsules loaded with curcumin (Nano-curcumin) were synthesized via nanoprecipitation and characterized for encapsulation efficiency, particle size, polydispersity, zeta potential, morphology, and curcumin release. Biocompatibility was assessed using rabbit oral mucosal cells via MTT and trypan blue assays. Antimicrobial efficacy was tested in vitro on primary dentin slices contaminated with S. mutans across four groups: Nano-curcumin, Nano-curcumin + PDT, diode laser, and untreated control. Colony-forming units (CFU) were quantified after treatment. Statistical analysis was performed using ANOVA and Tukey’s test (p < 0.05).

**Results:**

Nano-curcumin demonstrated high encapsulation efficiency (~100%), spherical morphology, low polydispersity (0.108), and favorable colloidal stability, with sustained curcumin release over 24 hours. Cytotoxicity assays showed >50% cell viability at 100 μg·mL⁻¹ and ~80% at intermediate concentrations (50–75 µg·mL^-^¹). Both curcumin nanocapsules and their photosensitized versions significantly reduced *S. mutans* CFU compared to controls (p < 0.05), with PDT-enhanced nanocapsules showing the greatest reduction, though not statistically different from non-photosensitized nanocapsules.

**Discussion:**

Curcumin-loaded zein nanocapsules are biocompatible and effective against *S. mutans*, with controlled release properties. Photodynamic activation further enhances antimicrobial activity, supporting their potential as a minimally invasive approach for managing carious lesions, particularly in pediatric dentistry. This strategy integrates a natural photosensitizer with a biodegradable polymeric matrix, providing a safe and innovative alternative for caries control.

## 1 Introduction

Dental caries continues to be a major public health issue, particularly among vulnerable populations. This is largely due to frequent carbohydrate consumption, increased acidity, and disruption of the oral microbiota (Takahashi and Nyvad, 2016). This acidic environment promotes pathogenic biofilm formation and enamel demineralization, with *Streptococcus. mutans* playing a central role. This bacterium efficiently metabolizes sugars, producing acid while thriving in low-pH conditions. It also contributes, to biofilm stability by synthesizing an extracellular polysaccharide matrix which enhances resistance to antimicrobial agents (de Oliveira et al., 2019; Pourhajibagher et al., 2019).

Although conventional treatments focus on removing caries and restoring teeth they often fall short in achieving long-term disease control. Primary teeth are especially susceptible to rapid caries progression due to their thinner dentin, larger pulp chambers, and increased permeability (Nehete et al., 2014). To address these challenges, minimally invasive techniques such as selective caries removal (SCR), stepwise caries removal (SWR), and the Hall Technique have been developed. These methods aim to preserve tooth vitality while minimizing patient discomfort (Aïem et al., 2020; Innes et al., 2011; Machiulskiene et al., 2020).

In addition to preserving tooth structure, controlling residual bacteria is essential to prevent pulp inflammation and recurrent lesion (Diniz et al., 2015). Photodynamic therapy (PDT) has emerged as a promising antimicrobial strategy, utilizing light-activated photosensitizers to selectively eliminate cariogenic bacteria (Wilson and Patterson, 2008). Among these, curcumin stands out as a photosensitizer due to its antibacterial (Carolina Alves et al., 2019), antifungal (Zorofchian Moghadamtousi et al., 2014), antineoplastic (Ghaffari et al., 2020), anti-inflammatory (Zhi et al., 2021) and antioxidant properties (Kamwilaisak et al., 2022).

When used in PDT, curcumin exhibits high cytotoxicity against pathogenic microorganisms, particularly against Gram-positive bacteria (Adamczak et al., 2020). These properties make it a promising candidate for the development of new antimicrobial therapies (Hosseinpour-Nader et al., 2022). Nanotechnology further enhances curcumin’s therapeutic potential by improving its stability, bioavailability, and antimicrobial efficacy (Hosseinpour-Nader et al., 2022). Incorporating nanoparticles into dental materials has also been shown to enhance their mechanical and biological properties (Andreatta et al., 2023; Batista et al., 2024; da Rosa et al., 2022; Masiero et al., 2024; Narciso et al., 2019; Parizzi et al., 2025).

In this context, targeted strategies against *S. mutans* including the use of nanoparticles to modulate the cariogenic microbiome have shown encouraging results (Sayed et al., 2020). However, despite growing interest in PDT and the the known antimicrobial potential of curcumin, few studies have explored the combined use of curcumin-loaded nanostructures and PDT in primary dentin, which differs morphological and histological characteristics compared to permanent teeth. Moreover, limited research has assessed the biocompatibility of such systems in healthy oral tissues, particularly in pediatric settings.

To address these gaps, the present study aimed to synthesize and characterize zein-based nanocapsules loaded with curcumin, evaluate their biocompatibility with oral mucosal cells, and investigate their their *in vitro* antimicrobial efficacy on primary dentin contaminated with *S. mutans*, both with and without photodynamic activation. This innovative approach combines a natural photosensitizers with a biodegradable polymeric matrix, offering a minimally invasive and potentially safer alternative for the treatment of carious lesions in children.

## 2 Material and methods

This study was approved by the Research Ethics Committee (CAAE No. 6.246.02).

### 2.1 Materials

The materials used in this study included curcumin, zein, poloxamer 407, and Dulbecco’s Modified Eagle Medium (DMEM), all from Sigma-Aldrich (Saint Louis, MO, USA). The culture media used comprised Mueller-Hinton agar and Tryptic Soy agar (Himedia, Thane, India), along with Brain and Heart Infusion Agar (BHI) (Merck, Darmstadt, Germany). The bacterial strain employed was *S. mutans* ATCC 25175. All other reagents were also obtained from Sigma-Aldrich.

### 2.2 Methods

#### 2.2.1 Synthesis and physicochemical characterization of zein nanocapsules loaded with curcumin

Curcumin-loaded zein nanocapsules (Nano-curcumin) were synthesized using the nanoprecipitation method in triplicate (n=3), following the methodology described by (Gonçalves da Rosa et al., 2020; Suzuki et al., 2016). To prepare the organic phase zein (20 mg mL^-1^) in 6.67 mL of 85% ethanol. Once fully solubilized, 134 μL of an alcoholic curcumin solution (1.5 mg mL^-1^) was added. The organic phase was then poured into 20 mL of an aqueous phase containing the surfactant Pluronic (0.8% v/v) under constant agitation at 10,000 rpm using a homogenizer IKA T25 homogenizer (IKA, Wilmington, NC,USA) for 3 minutes.

Nanocapsule formation occurred via nanoprecipitation upon contact between the two phases. The resulting suspension was stirred under a fume hood with magnetic agitation to ensure complete evaporation of the organic solvent. A control formulation without curcumin (Nano-curcumin-free) was prepared using the same procedure.

To confirm nanoencapsulation, the encapsulation efficiency (EE) of the curcumin-loaded zein nanocapsules was evaluated in triplicate (n=3) following to the methodology described by (Gonçalves da Rosa et al., 2020). EE was determined using a centrifugal ultrafiltration method, as outlined by Parizzi et al. (2025). Samples were centrifuged using Amicon Ultra centrifugal filters with a 30 kDa Ultracel membrane at 6,000 rpm for 30 minutes, allowing non-encapsulated curcumin to pass through the membrane.

The free curcumin in the supernatant was quantified using UV-Vis spectroscopy (Spectrostar Nano, BMG Labtech, Weston Parkway Suite, NC, USA) at a wavelength of approximately 430 nm. The molar concentration of curcumin was calculated based on a calibration curve prepared with an alcoholic curcumin solution.

Encapsulation efficiency (EE) was calculated using Equation 1:


(1) $$EE\%=\frac{[\left(initial\;curcumin\;-\;free\;curcumin\right)}{\left(initial\;curcumin\right)]\;x\;100}$$

To confirm curcumin encapsulation, UV-Vis spectrophotometry was performed using a Spectrostar Nano scanning spectrophotometer (BMG Labtech, Weston Parkway Suite, NC, USA). Measurements were taken across a wavelength range of 200 to 600 nm, with a resolution of 1 nm. The absorbance peak (λmax) of free curcumin was determined after dilution in absolute ethanol, while nanocapsule suspensions were diluted in ultrapure water prior to analysis.

Physicochemical characterization included the assessment of particle size (nm), polydispersity index (PDI), and zeta potential (mV), using dynamic light scattering (DLS) with a Zetasizer Advance (Malvern Panalytical, Worcestershire, UK). Samples of Nano-curcumin and control formulations were diluted in Milli-Q^®^ water and analyzed at 25 °C, with a scattering angle of 173°, in triplicate (n = 3), using electrophoretic cells.

Nanocapsule morphology was examined using transmission electron microscopy (TEM) JEOL JEM 2100 (Tokyo, Japan) operating at 70 kV. Solutions containing curcumin-loaded zein nanocapsules and control samples were diluted in ultrapure Milli-Q water. Approximately 5 µL of each sample was deposited onto carbon-coated copper grids (200 mesh). After air drying at room temperature, the grids were observed under the microscope.

The curcumin release assay was conducted using a citrate–phosphate buffer at pH 7.0, as described by Parizzi et al. (2025). For each experiment, 1 mL of the nanoparticle dispersion was placed into a dialysis membrane (pore size: 25 Å; molecular weight cut-off: 12,000–16,000 Da) and immersed in 100 mL of buffer under continuous stirring. Samples of the external medium were collected at regular intervals from 1 to 8 hours, with additional aliquots taken at 12 and 24 hours. The amount of curcumin released was quantified by UV-Vis spectrophotometry using the Spectrostar Nano (BMG Labtech, Weston Parkway Suite, NC, USA) at 425 nm. Concentrations were determined using a calibration curve constructed with curcumin standards.

#### 2.2.2 Cytotoxicity and cell viability assay

The cytotoxicity and cell viability of curcumin-loaded zein nanocapsules (Nano-curcumin) were evaluated using surface mucosal cells derived from rabbits. The cells were cultured in high-glucose Dulbecco’s Modified Eagle Medium (DMEM) supplemented with 10% fetal bovine serum, 100 U/mL penicillin, and 100 mg/mL streptomycin. Cultures were maintained in a humidified atmosphere at 37 °C with 5% CO_2_ and 95% air until confluence was reached.

Once confluent,the cells were seeded at a density of 10,000 cells per well in 96-well plates. A single dose of nanocapsules was added at concentrations of 25, 50, 75, and 100 μg·mL^-1^.

Following treatment, the samples were irradiated using a low-power diode laser InGaAlP (DMC-Therapy, Sao Carlos, SP, Brazil) at a wavelength of 660 nm, continuous emission, a power output of 100 mW, and a total energy of 9 joules over 90 seconds (Knorst et al., 2019).The laser tip was positioned 10 mm above the wells, and irradiation was applied alternately to sets of four wells to ensure proper spacing between the light source and avoid overlapping light exposure.

Cell viability was assessed using the MTT assay (0.5 mg·mL^-1^) and trypan blue exclusion (TBE). For the MTT assay, 20 μL of a stock MTT solution (5 mg·mL^-1^) was added to each well and incubated for 4 hours. After incubation, cells were dissolved in DMSO, and optical density was measured at 490 nm in equipment Spectrostar Nano (BMG Labtech, Weston Parkway Suite NC, USA)The percentage of viable cells relative to the control was calculated based on the absorbance values, considering the ratiobetween treated cells (Abs. sample) and the absorbance of the cell-free culture medium (Abs blank), as indicated in Equation 2.


(2) Cell viability (%)= (Abs. sample)/(Abs.blank) x 100

#### 2.2.3 *In vitro* antimicrobial evaluation of zein nanocapsules loaded with curcumin on primary tooth dentin contaminated with *Streptococcus mutans*

##### 2.2.3.1 Sample preparation

Forty mandibular and maxillary primary molars free of caries, restoration and with no visible cracks or fractures were collected for this study. The teeth were donated by patients prior consent from their legal guardians. After collection, the specimens were washed to remove impurities, sterilized in an autoclave, and stored in distilled water until sectioning. For the cutting procedure, each tooth was mounted on an acrylic plate using low-melting-point plasticized wax and sectioned using a precision cutting machine (Isomet 1000, Buehler, Coventry, UK). Sections of approximately 1 mm thick were obtained using a 0.4 mm diamond disc (Buehler, Lake Bluff, IL, USA) operating at 300 rpm. Based on the inclusion criteria a final sample of 28 slices was selected. These slices were then sterilized again by autoclaving before being used in the contamination procedure.

##### 2.2.3.2 Bacterial culture and contamination procedure

To evaluate the antimicrobial activity of the nanoparticles, lyophilized *S. mutans* (ATCC 25175) strains were rehydrated according to the manufacturer’s instructions and incubated anaerobically in Tryptone Soy Broth (TSB) at 37°C for 48 hours. Following incubation, the samples were plated on solid Blood Agar using the streak plate method to obtain isolated colonies. From these colonies, a bacterial suspension equivalent to 1.5 × 10^8^ cells·mL^-^¹ was prepared using the 0.5 McFarland scale. The 1 mm dentin slices were then incubated in 990 μL of TSB medium supplemented with 10 μL of the *S. mutans* bacterial suspension and maintained under anaerobic conditions at 37°C for 48 hours.

##### 2.2.3.3 Experimental groups and treatments

After the incubation period, the dentin slices were removed from the bacterial suspension, transferred to 1 mL of saline solution, and immediately divided into the following experimental groups (n = 7):

- Group 1: Contaminated Dentin + NanoCurcumin (Dent-NanoCurcumin)
- Group 2: Contaminated Dentin + NanoCurcumin + Photodynamic Therapy (Dent-NanoCurc-PDT)
- Group 3: Contaminated Dentin + Diode Laser
- Group 4: Contaminated Dentin (Dent) – Control

Groups 1 and 2 were incubated with 1 mL of their respective nanocapsule dispersion (containing 7.5 µg/mL of curcumin) for 4 hours at room temperature, while Groups 3 and 4 were incubated with saline solution under the same conditions. Subsequently, Groups 2 and 3 were treated with a low-power InGaAlP diode laser (DMC-Therapy, São Carlos, SP, Brazil) at a wavelength of 660 nm, in continuous emission mode, with a power output of 100 mW and a total energy of 9 joules applied over 90 seconds (Parizzi et al., 2025; Knorst et al., 2019).

##### 2.2.3.4 Microbiological Analysis

Following the treatments, the dentin slices were transferred to 1 mL of saline solution and immediately incubated in TSB broth for 30 minutes. To assess antimicrobial activity, 10 μL of the broth was placed at the center of a sterile Petri dish, over which Mueller-Hinton agar was poured. After solidification, the plates were incubated anaerobically at 37°C for 48 hours. Colony-forming units (CFU) were then counted, and the results were expressed as CFU·mL^-^¹ (Fernandes et al., 2022).

##### 2.2.3.5 Data analysis

Results were expressed as means and standard deviations from triplicate measurements. Statistical analysis was performed using analysis of variance (ANOVA), followed by Tukey’s test for multiple comparisons, with a significance level of 5%. Data were analyzed using STATISTICA 7 software.

## 3 Results

### 3.1 Physicochemical characterization of nanocapsules

**Figure 1:**
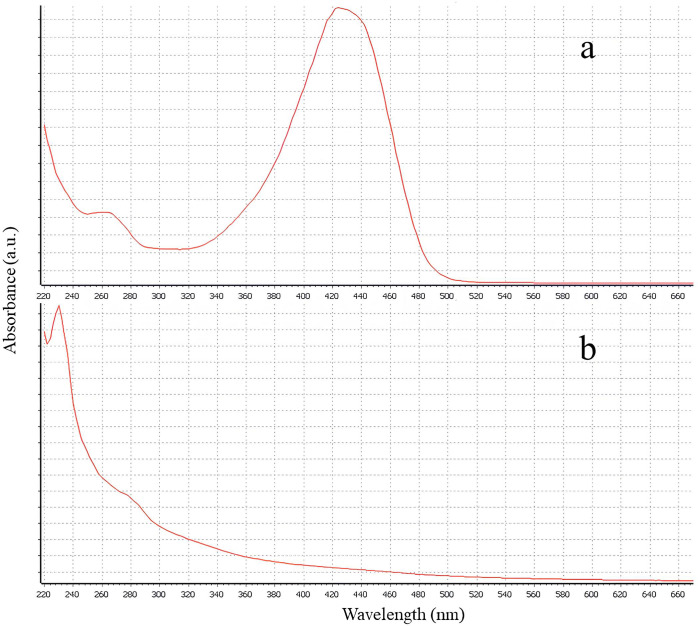
UV-Vis scan spectra: **(a)** free curcumin; **(b)** nanocurcumin.

The encapsulation efficiency of curcumin in zein matrices was close to 100%. **Figure 1** shows the absorbance spectra of free curcumin and nanocurcumin, obtained by UV-Vis spectroscopy. Free curcumin exhibited a well-defined absorbance peak at approximately 425 nm. In contrast, the nanocurcumin spectrum displayed an altered profile, with the absence of this characteristic peak and increased absorbance in the UV region.

**Figure 2:**
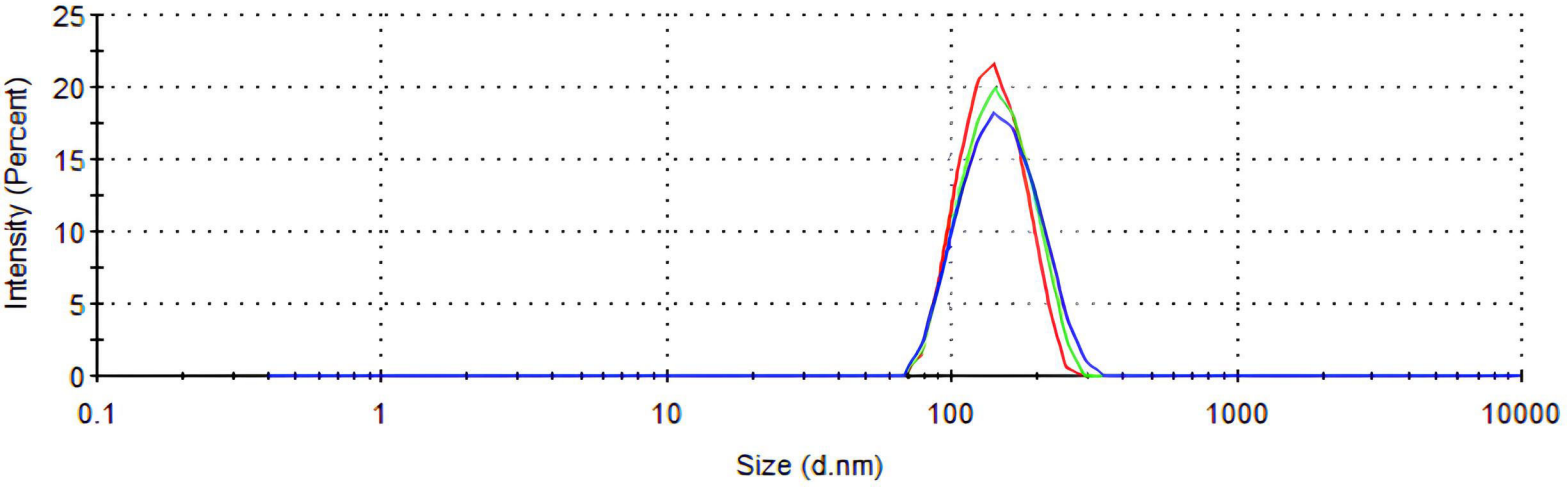
Particle size distribution of the Nano-curcumin sample.

**Table 1** presents the measurements of average particle size, polydispersity index (PDI), and zeta potential for nano-curcumin and nano-curcumin-free samples. Nano-curcumin particles exhibited a larger average size (139.4 ± 1.0 nm) compared to the nano-curcumin-free particles (128.5 ± 0.7 nm), with a statistically significant difference (**Figure 2**). The PDI values indicate that nano-curcumin had a lower polydispersity index (0.108 ± 0.07) whereas nano-curcumin-free showed a significantly higher PDI (0.271 ± 0.03). Regarding zeta potential, nano-curcumin exhibited a lower value (10.9 ± 0.5 mV) compared to nano-curcumin-free (40.0 ± 2.8 mV), also with a significant difference.

**Table 1:** Average particle size, polydispersity index (PDI), and zeta potential.

Sample	Average size (nm)	Polydispersity index (PDI)	Zeta potential (mV)
Nano-curcumin	139.4 ± 1.0a	0.108 ± 0.07b	10.9 ± 0.5b
Nano-curcumin-free	128.5 ± 0.7b	0.271 ± 0.03a	40.0 ± 2.8a

Results are expressed as mean ± standard deviation (n=3). Different letters indicate significant differences (p<0.05) when analyzed by Tukey’s test within the column.

**Figure 3:**
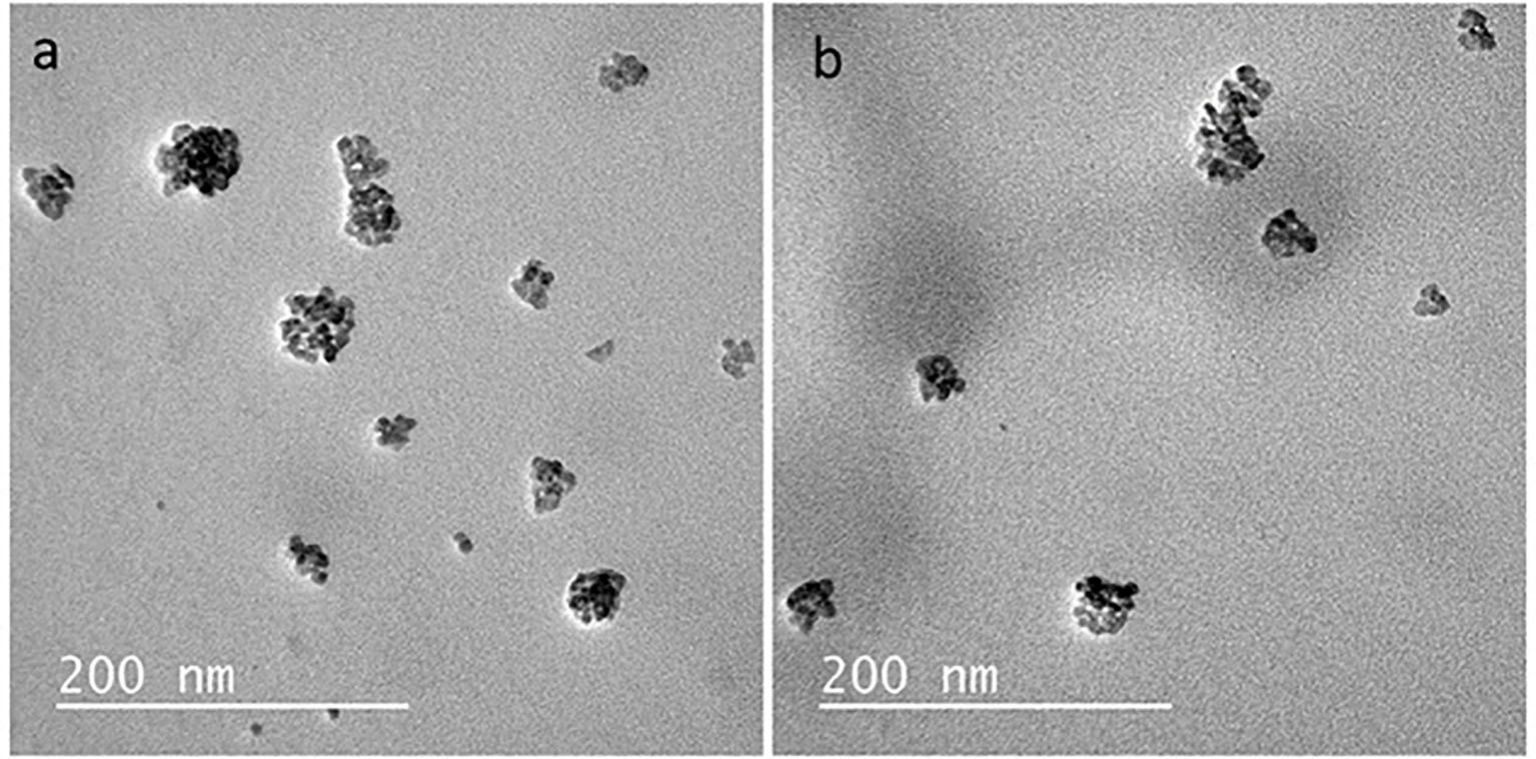
TEM micrographs of the nanocapsules: **(a)** Nano-curcumin-free **(b)** Nano-curcumin.

The transmission electron microscopy (TEM) analysis of zein nanocapsules loaded with curcumin revealed key morphological characteristics. TEM micrographs indicated that the nanocapsules exhibited a spherical shape (**Figure 3**).

**Figure 4:**
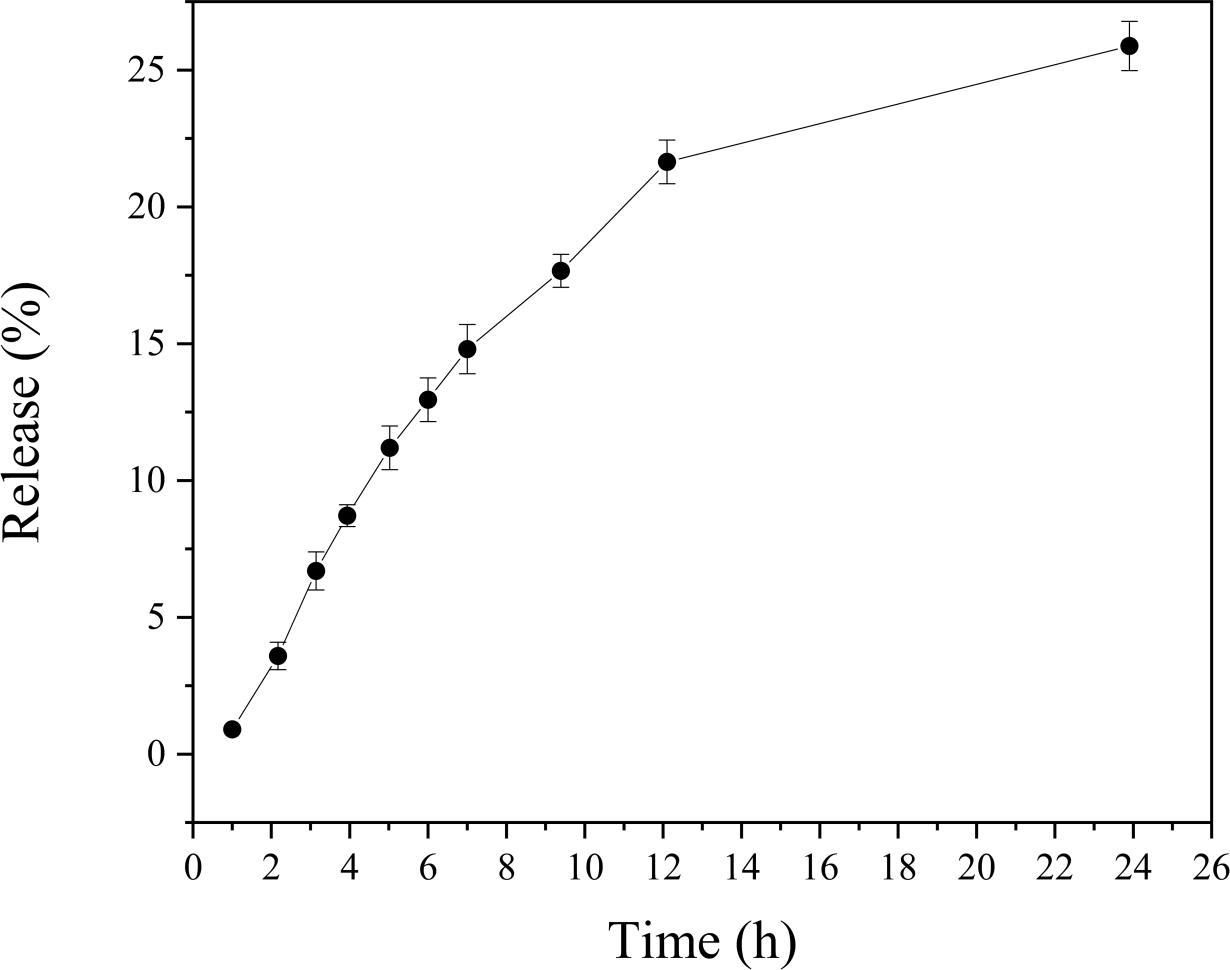
Release of nano-curcumin in citrate–phosphate buffer (pH 7.0) over 24 hours.

Spectrophotometric analysis showed that nano-curcumin exhibited a sustained release in buffered aqueous medium over a 24-hour period. The release profile indicates a gradual increase in curcumin concentration in the external medium, with a more pronounced release during the first 8 hours and a tendency toward stabilization after 12 hours, as shown in **Figure 4**.

### 3.2 Cytotoxicity and cell viability assay

**Figure 5:**
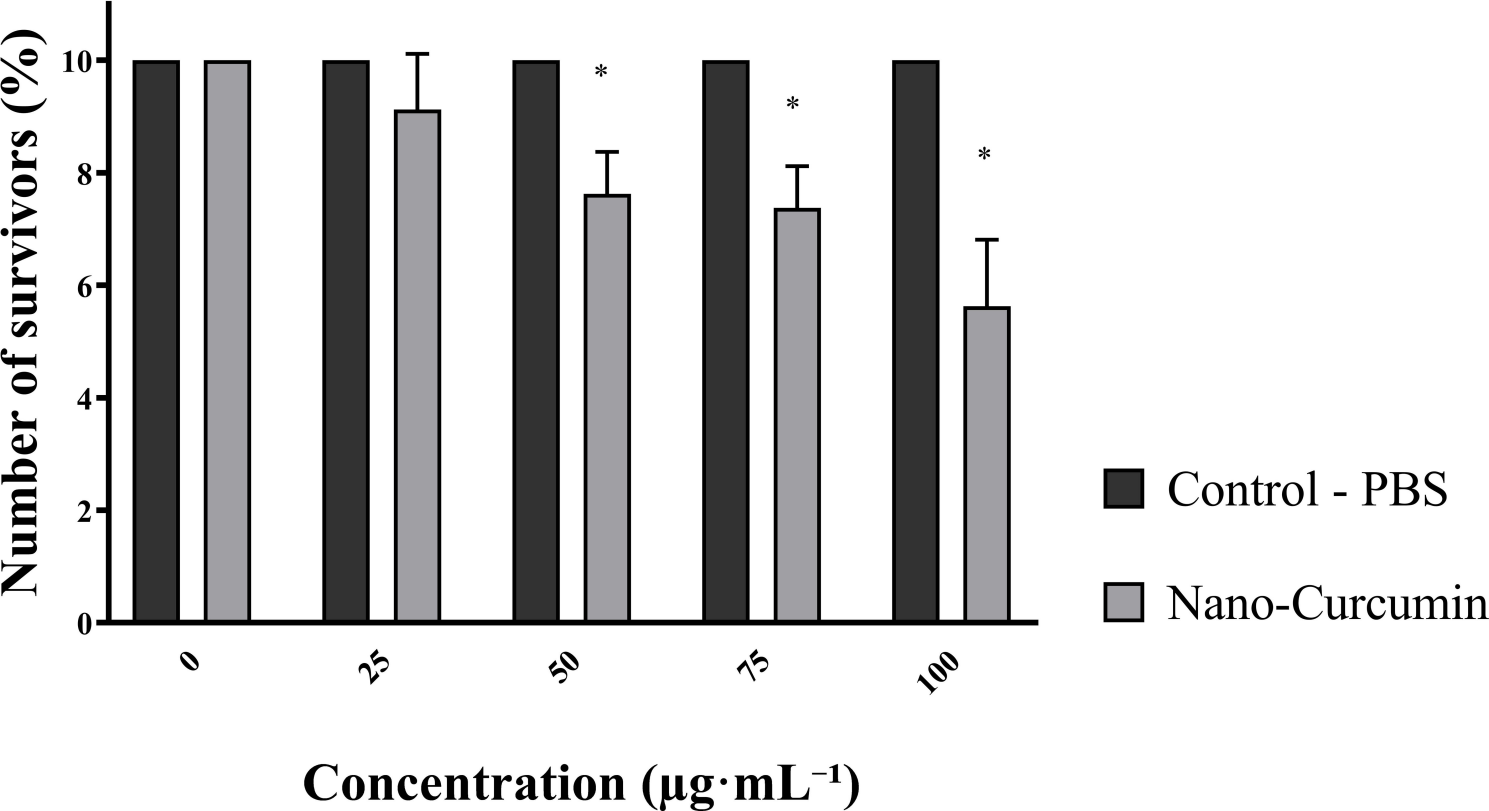
Survival of oral mucosal cells in response to different concentrations of zein nanocapsules loaded with curcumin. *The p-values: p < 0.05 indicate a significant difference according to Tukey’s test.

At a 100 μg·mL-1 nanocapsule concentration, the cell survival rate exceeded 50μg·mL-1 while concentrations of 50 μg·mL-1 and 75μg·mL-1 resulted in cell survival rates approaching 80% (**Figure 5**).

**Figure 6:**
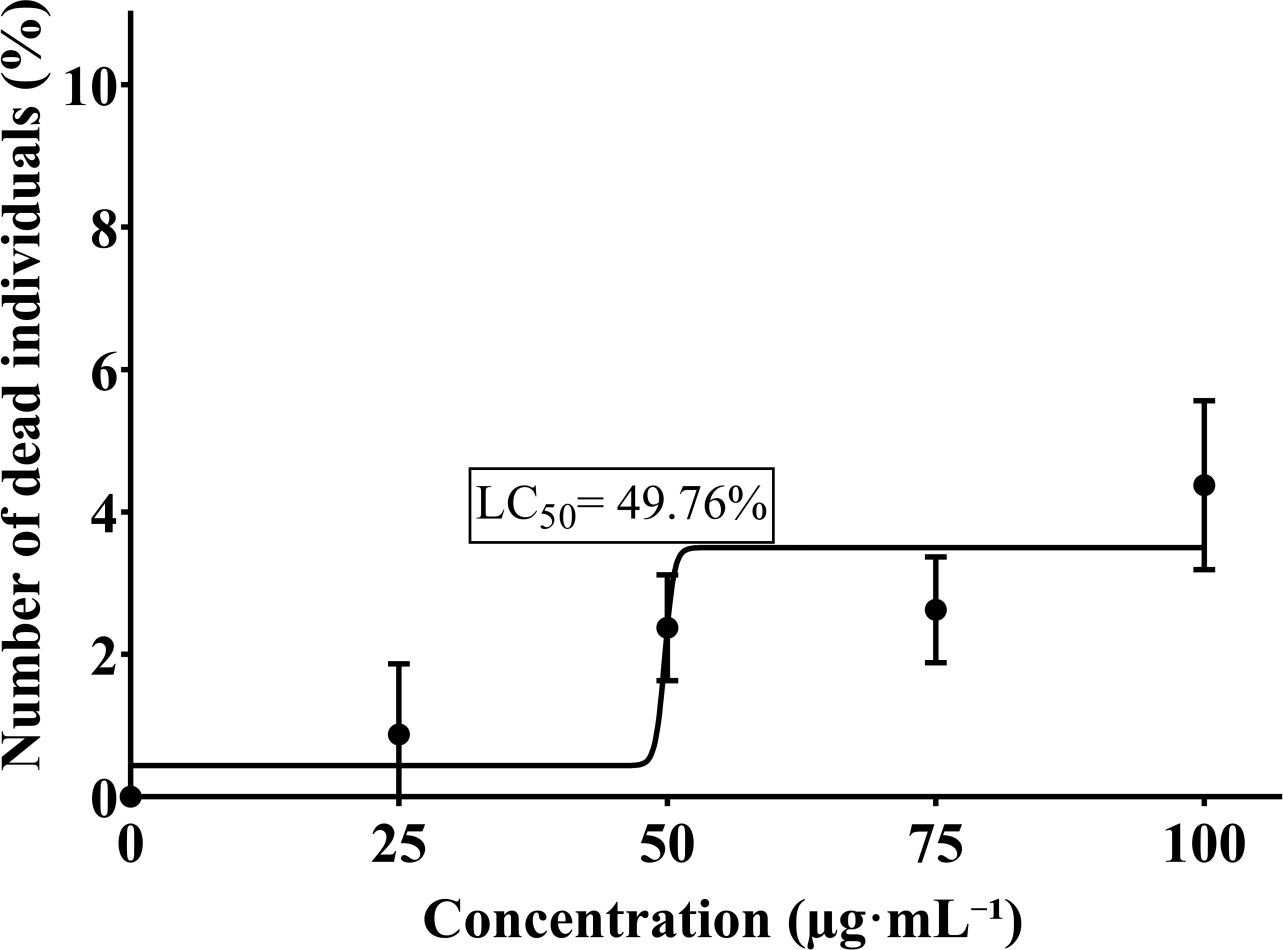
Median lethal concentration (LC50) of zein nanocapsules loaded with curcumin.

Zein nanocapsules loaded with curcumin at a 100 μg·mL-1 concentration (7.5 µg·mL^-^¹ of curcumin), indicating that half of the healthy oral mucosal cells exposed to this concentration did not survive (**Figure 6**).

### 3.3 Microbiological analysis

**Figure 7:**
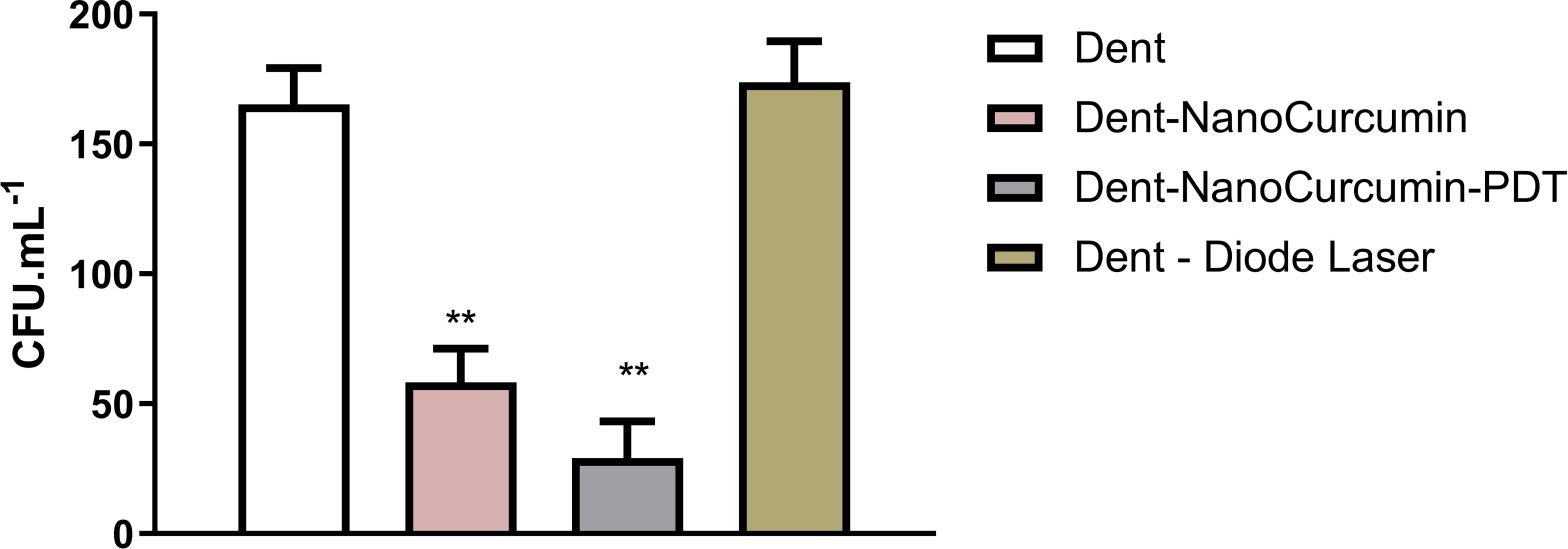
Comparison of antimicrobial effectiveness against s. mutans between the tested groups. **The p-values: p < 0.05 indicate a significant difference according to Tukey’s test.

The results of the microbiological analysis of dentin contaminated with *S. mutans* are presented in **Figure 7**. Both curcumin nanocapsules and their photosensitized versions significantly reduced *S. mutans* CFU/mL compared to untreated controls (p<0.05). However, the group treated with photosensitized curcumin nanocapsules exhibited the lowest CFU/mL count, this reduction was not statistically different from that observed in the non-photosensitized curcumin nanocapsules group.

## 4 Discussion

The high encapsulation efficiency (~100%) of curcumin in zein matrices observed in this study aligns with previous reports highlighting the effectiveness of this biopolymer as a carrier for bioactive compounds (Da Rosa et al., 2020). Given curcumin’s poor solubility and susceptibility to degradation (Hu et al., 2024), encapsulation within a zein matrix offers significant advantages including enhanced stability, protection against oxidation, and shielding from adverse interactions that could compromise its biological activity in the oral environment (Choi et al., 2016). This high retention capacity is particularly advantageous for controlled-release systems, where sustained bioavailability at the target site—such as infected dentin—is essential for therapeutic efficacy.

UV-Vis spectroscopy revealed notable changes in the absorbance profile of curcumin following encapsulation. Free curcumin exhibited a distinct peak at approximately 425 nm, corresponding to π→π* electronic transitions of the β-diketone conjugated system in its enolic form, typically observed in organic solvents (Urošević et al., 2022). In the nanoencapsulated form, this peak was significantly diminished or absent, with a relative increase in absorbance at wavelengths below 400 nm (Wu et al., 2023). These spectral changes suggest that curcumin was incorporated into the hydrophobic regions of the polymeric matrix, resulting in conformational restriction and reduced interaction with the dispersion medium.

These modifications are likely due to non-covalent interactions between curcumin and zein. Zein contains hydrophobic segments that facilitate molecular entrapment and enable additional intermolecular interactions, such as hydrogen bonding and Van der Waals forces (Chen et al., 2015). Furthermore, π–π stacking between the aromatic rings of curcumin and zein contributes to the structural stabilization of the nanoparticles (Ding et al., 2023; Liu et al., 2023).

In addition to confirming encapsulation, comparative studies have shown that encapsulated curcumin undergoes significantly less degradation under UV radiation. Literature indicates that free curcumin degrades by more than 60% after 30 minutes of exposure, whereas the encapsulated form shows less than 10% degradation under the same conditions (Wu et al., 2023). Encapsulation also promotes the dispersion of curcumin in an amorphous state, enhancing its solubility in aqueous media and absorption in the gastrointestinal tract, potentially improving oral bioavailability (Liu et al., 2023).

Thus, the attenuation of the 425 nm peak and the altered absorbance pattern observed in nano-curcumin reflect structural constraints imposed by the zein matrix, resulting in increased protection against degradation and greater potential for use in nutraceutical and pharmaceutical formulations.

The observed increase in nanoparticle size following curcumin incorporation suggests strong physicochemical interactions between zein and curcumin during nanoprecipitation process. This contributes to greater structural stability and reduced aggregation, attributed to the hydrophobic nature of curcumin (Da Rosa et al., 2015). Additionally, the lower polydispersity index (PDI) observed in curcumin-loaded formulations compared to those without the compound indicates a more uniform size distribution - an important factor for optimizing bioavailability and ensuring effective dentin penetrationthereby enhancing antimicrobial action against cariogenic microorganisms (Danaei et al., 2018).

Zeta potential results further support these findings. The lower surface charge observed in nano-curcumin formulations suggests potential alterations in colloidal stability due to interactions with zein. While higher zeta potential values typically indicate greater electrostatic stability (Nunes et al., 2022), the reduction observed here may increase the likelihood of aggregation over time, emphasizing the need for appropriate stabilization or storage strategies to preserve therapeutic efficacy (Danaei et al., 2018).

These findings underscore the potential of nano-curcumin formulations for targeted antimicrobial therapy in dentistry, particularly in minimally invasive approaches for caries management. By ensuring high encapsulation efficiency, enhanced stability, and controlled release, this formulation presents a promising alternative for dental applications. Future studies should explore its long-term stability and *in vivo* performance to validate clinical applicability and optimize formulation parameters.

The sustained release observed is consistent with the release mechanisms commonly associated with zein-based polymeric systems. Due to its hydrophobic and compact structure, zein acts as a physical barrier to drug diffusion, promoting a passive and controlled release profile (Oh and Flanagan, 2010; Gonzalez-Valdivieso et al., 2021). Hydrophobic interactions and potential hydrogen bonding between curcumin and the zein matrix further contribute to compound retention, limiting its rapid diffusion into the external medium (Lenzuni et al., 2023; Zhang et al., 2023).

Curcumin’s inherently low aqueous solubility (<0.001 mg/mL) further restricts its release in buffered aqueous environments. Even after encapsulation, this intrinsic property limits immediate availability, reinforcing the gradual release profile observed (Karthikeyan et al., 2020; Omidian et al., 2023). The combination of poor solubility and the hydrophobic nature of zein results in a controlled release without burst effects.

The release of bioactive compounds from zein-based systems typically follows anomalous kinetics, often fitting the Korsmeyer–Peppas model. This suggests that both diffusion and slow matrix relaxation or degradation contribute to the release mechanism (Zhang et al., 2021; Lin et al., 2024). These processes occur simultaneously, with diffusion predominating in the initial phase and matrix reorganization influencing release behavior at later stages.

Systems with these characteristics are highly desirable for topical formulations targeting the oral cavity. Prolonged release on surfaces such as the oral mucosa, gingiva, or dentin enhances the local retention of curcumin, thereby amplifying its pharmacological activity. Curcumin is well known for its anti-inflammatory, antioxidant, and antimicrobial properties, with demonstrated benefits in the treatment of gingivitis, periodontitis, and chronic oral lesions (Inchingolo et al., 2024). Moreover, controlled release reduces the frequency of reapplication and contributes to a superior safety profile by avoiding local concentration peaks. These findings confirm that zein is an effective carrier system for the controlled release of hydrophobic compounds like curcumin, representing a promising strategy for minimally invasive therapies in oral healthcare.

The results of this study confirm the biocompatibility of zein nanocapsules loaded with curcumin for oral mucosal cells—an essential requirement for safe use in dental applications. Minimizing cytotoxicity is Inchingly critical to ensure that formulation does not harm surrounding healthy tissues while maintaining its therapeutic effects (Shahi et al., 2019). In this study, at the highest tested concentration of nanocapsules (100 μg·mL^-^¹, equivalent to 7.5 μg·mL^-^¹ of curcumin), cell survival remained above 50%. At concentrations of 50 and 75 μg·mL^-^¹, viability approached 80%. Although a viability threshold above 50% is generally acceptable, these results suggest that even at full concentration, the formulation does not induce critical toxicity. This is particularly relevant for applications in the oral cavity, where direct mucosal exposure requires safe and well-tolerated materials.

The observed LC_50_ value (49.75 µg·mL^-^¹) indicates that 50% of the exposed cells did not survive at the highest tested concentration. LC_50_ is a standard toxicological parameter representing concentration at which half of the cells are affected, serving as a key indicator of formulation safety (Idrees and Kujan, 2023). The nanometric scale of the zein nanocapsules may have enhanced their penetration and interaction with oral mucosal cells, increasing curcumin’s local bioavailability. While this property is advantageous for targeted antimicrobial action, it also underscores the importance of carefully optimizing dosage to mitigate toxicity risks and maintain a favorable therapeutic index.

These findings are consistent with previous studies on the Inchingly biocompatibility of curcumin when encapsulated in nanocarriers. Meng et al. (2023) demonstrated that starch-based nanocapsules loaded with curcumin exhibited low toxicity toward healthy cells while effectively inhibiting tumor cells, suggesting that encapsulation plays an important role in modulating curcumin’s biological interactions. Similarly, Minhaco et al. (2023) reported over 80% cell viability at 325 μg/mL using PLGA-curcumin nanoparticles in oral cells, supporting their biomedical potential.

While reducing zein-curcumin concentrations may lower cytotoxicity, maintaining antimicrobial efficacy is key. Curcumin nanostructures remain effective at low doses; for example, 6.25 µg/mL of curcumin nanocrystals inhibited P. gingivalis growth. The 7.5 µg/mL used in this study supports the efficacy of nano-curcumin at low concentrations (Maleki Dizaj et al., 2022).

These results reinforce the promise of zein-based curcumin nanocapsules for safe, effective dental applications—especially in pediatric care, where non-invasive, biocompatible treatments are preferred.

Photodynamic therapy (PDT) with photosensitizers (PS) is well-documented for treating oral pathogens, including *S. mutans* (Cusicanqui Méndez et al., 2019; Hosseinpour-Nader et al., 2022; Manoil et al., 2014; Parizzi et al., 2025; Silvestre et al., 2023). Upon irradiation, the PS generates reactive oxygen species (ROS), primarily singlet oxygen, which promotes oxidative damage to microbial DNA, organelles, and cell membranes, ultimately leading to bacterial death (Taraszkiewicz et al., 2013).

Curcumin, a natural polyphenol derived from plants, displays broad-spectrum antibacterial activity due to its unique molecular structure and antioxidant properties. It disrupts quorum sensing, impairs biofilm formation, reduces virulence factor expression, and prevents bacterial adhesion to host cell receptors. When activated, curcumin acts as a photosensitizer, generating phototoxic effects that inhibit bacterial growth and enhance the efficacy of other antimicrobials through synergistic interactions (Zheng et al., 2020).

By producing reactive oxygen species (ROS) such as hydrogen peroxide, superoxide, and singlet oxygen, curcumin damages bacterial cell structures by oxidizing membranes, proteins, and nucleic acids, leading to cell death. Gram-positive bacteria are particularly susceptible due to their permeable peptidoglycan-rich cell walls, which facilitate photosensitizer entry (Ghate et al., 2019).

Additionally, curcumin induces intracellular damage, affecting DNA and proteins, disrupting biofilm adhesion, and downregulating virulence genes involved in pathogenicity. While its effect on outer membranes is limited, photodynamic inactivation (PDI) with curcumin causes significant internal damage and cytoplasmic leakage. The superior antimicrobial effect of light-activated curcumin highlights its potential for targeting biofilm-associated infections and oral pathogens such as *Streptococcus mutans* (Ghate et al., 2015; Hu et al., 2018; Huang et al., 2020; Pereira et al., 2014; Tonon et al., 2015).

Microbiological analysis confirmed that both conventional and photosensitized curcumin nanocapsules significantly reduced *S. mutans* CFU/mL in dentin. Although the photosensitized group showed the lowest count, the difference was not statistically significant.

This may be due to the strong inherent antimicrobial activity of nano-curcumin, suboptimal PDT parameters (e.g., light dose, wavelength, pre-irradiation time), or limited light penetration into biofilms. Pre-irradiation time is crucial for PS penetration (de Oliveira et al., 2019), especially in mature biofilms, which are more resistant due to their dense extracellular matrix (Silvestre et al., 2023; Taraszkiewicz et al., 2013).

In this study, extended pre-irradiation was used to assess whether PDT efficacy could be enhanced or if nano-curcumin alone was sufficient. Less structured biofilms may respond well to nano-curcumin without PDT, as the formulation improves curcumin delivery and release.

Curcumin’s poor solubility and rapid degradation limit its therapeutic use, but nanoencapsulation improves stability, lowers the MIC, and enhances antimicrobial efficacy (Pourhajibagher et al., 2018).

Studies support combining PDT with nano-curcumin. Pourhajibagher et al. (2022) found that a 5% nano-curcumin cavity liner with PDT inhibited *S. mutans* for 60 days. Araújo et al. (2017) required 5 g·L^-^¹ for photoactivation effects on *S. mutans* and *L. acidophilus*. In contrast, this study used only 0.0075 g·L^-^¹, yet showed efficacy—suggesting nanoencapsulation allows lower doses and reduces side effects like dental staining.

PDT also improves restoration outcomes.Clinical studies show its use in selective caries removal (SCR) reduces microbial load and enhances restoration success (Alves et al., 2019; Borges et al., 2010; Melo et al., 2015; Steiner-Oliveira et al., 2015). Long-term data confirm no compromise in restoration integrity after 6–12 months (Alves et al., 2019).

Combining nano-curcumin with PDT promotes remineralization by enhancing calcium and phosphate penetration into demineralized dentin (Koo et al., 2013; Wilson and Patterson, 2008; Zaleh et al., 2022). This approach aligns with minimally invasive dentistry, especially in pediatrics, by preserving primary teeth, reducing discomfort, and shortening treatment time. However, PDT is less effective in deeper dentin due to light scattering and absorption (Koo et al., 2013; Zanin et al., 2005), highlighting the need for personalized treatment based on lesion depth.

Despite promising results, limitations exist. *In vitro* conditions don’t fully replicate the oral environment, where saliva, mechanical forces, and microbial diversity affect outcomes. While effective against *S. mutans*, further studies should assess polymicrobial biofilms. Curcumin’s limited dentin penetration may reduce efficacy in deep lesions, emphasizing the need to optimize nanoencapsulation for better bioavailability.

Tooth staining is another concern. Although some *in vitro* studies report no significant discoloration (Araujo et al., 2023), these are based on simplified models. PDT with nano-curcumin may be a viable alternative to silver diamine fluoride, especially in posterior teeth where esthetics are less critical (Araujo et al., 2023).

Long-term *in vivo* studies are needed to assess safety, biocompatibility, and clinical effectiveness. Future research should refine PDT parameters—pre-irradiation time, light dose, and treatment frequency—and explore nano-curcumin’s role in remineralization and its interaction with restorative materials.

## 5 Conclusions

This study synthesized and characterized curcumin-loaded zein nanocapsules with high encapsulation efficiency, spherical morphology, low polydispersity, and good colloidal stability. Cytotoxicity assays showed oral mucosal cell viability above 50% at high concentrations, supporting safety for topical use. Curcumin nanocapsules significantly reduced *S. mutans* on primary dentin. Although PDT further reduced bacterial load, the difference was not statistically significant, indicating the nanocarrier alone enhances antimicrobial efficacy via improved penetration and sustained release. With the limitation of this study, these results support nano-curcumin as a safe, effective, and minimally invasive strategy for caries management in primary teeth, aligning with conservative pediatric dental practices.

## Data Availability

The original contributions presented in the study are included in the article/supplementary material. Further inquiries can be directed to the corresponding author.
